# The never-ending process of adaptation

**DOI:** 10.1051/ject/2026005

**Published:** 2026-03-13

**Authors:** Luc Puis

Perfusionists typically perform the task of substituting the function of heart and lungs by means of cardiopulmonary bypass. In doing so, different organs of the body are affected as well, and one of those is the brain. The brain is a marvelous, complex structure, and recent discoveries reveal ever-growing insights into its function and how it is being influenced by what is happening in the rest of our body and vice versa.

While it was long thought that the brain was a static entity, we now know that it continually undertakes functional and structural reorganizations to adapt itself to influences from outside, referred to as neuroplasticity. Occupational neuroplasticity is a branch of that science that relates to neuroplasticity influenced by the daily work and activities that we do [1]. Through advanced imaging techniques, researchers have discovered that those functional changes happen in specific regions of the brain [1, 2], and that structural reorganization is dependent on the complexity of, and the experience with the tasks related to the occupation [3]. Literally, more experienced perfusionists have more gray matter. Unfortunately, it is also seen that prolonged unhealthy stress and malpractice can lead to maladaptive changes, resulting in pathologic conditions [1, 2]. In that perspective, neuroplasticity almost behaves like the immune system, which protects us, but can lead to pathologic conditions in a similar way, when overstimulated or uninhibited.

Knowing that, we can assume that perfusionists, who are constantly adapting to new techniques, lifelong learning and especially, acute changes in a patient condition, must have a high degree of occupational neuroplasticity. But that also means that our occupation is equally exposed to a higher risk of maladaptation. Perfusionists must adapt to each patient they treat and to each changing situation they are confronted with. It is shown that the forementioned volume increase in gray matter, is already apparent after only two practice sessions of a complex task [4].

Therefore, it is not surprising that several manuscripts in this edition of the Journal of ExtraCorporeal Technology are forcing you to adapt to new thinking and/or were the result of clinicians having to adapt to patients’ needs. While you are reading this editorial and the almost one hundred pages of featured articles from the USA, Mexico and Argentina, a lot of neuroplasticity is hopefully being carried out. We have a good number of papers dealing with the pediatric part of our patient population, and pediatric perfusionists are perhaps even more subjected to adaptation.

A prime example of having to adapt to patients’ needs is the case report [5] of surgery on a neonate with a combination of two very rare congenital cardiac conditions. An interrupted aortic arch (IAA) combined with an aortopulmonary window (APW). What is unique in this manuscript, is that one congenital defect was used to make the repair of the other defect easier.

In most IAA repairs, a second arterial cannula in the patent ductus arteriosus is needed for circulation of the lower body. In this case however, thanks to the presence of the APW, which was utilized as a direct conduit to the descending aorta through the patent ductus arteriosus, it allowed the use of a single arterial cannula for perfusion [5].

An equally fine example of adapting to patients’ presentations is a case report describing a complicated tracheoesophageal fistula repair [6] which had the surgical and ECMO teams thinking outside the box. By using veno-venous ECMO intraoperatively, and a Montgomery T-tube postoperatively during this challenging, 10-hour long case, they allowed surgical manipulation of mediastinal structures during apnea, ensured adequate gas exchange in the absence of mechanical ventilation, and enhanced comfort during postoperative splinting of the repaired trachea. Of note, is that the perfusionist running the ECMO was ELSO-certified, highlighting the advantages of continuous learning and certification [6]. That lifestyle of pursuing mental stimulation is protective against the decline of your brains’ flexibility and offers a “cognitive reserve” that will shield you from the disadvantages of ageing [2].

While the other submissions featured in this edition all have something to do with adaptation to novel insights, challenging patient cases, and progressing perfusion techniques, one article, solely authored by Kara Lung, CCP of Boston Children’s Hospital, deserves your special attention, as it offers staggering insights into how we as perfusionists (and other healthcare workers), need to learn new ways of dealing with data and protect patient health records from cybercrime. While this is a subject that seems to be a long way from the core tasks we practice daily, it is baffling to hear that employee carelessness is the cause of about 70% of all data breaches in general, and particularly in healthcare, human error accounts to about 95% of data breaches [7].

With ever-growing interconnectivity across devices, which makes it often easier and safer to treat patients, comes the tradeoff of a growing vulnerability of the data that is being collected on a patient’s condition. Not only does this extensive manuscript provide a historical overview of healthcare cybersecurity, but it also offers specific recommendations for perfusion teams that wish to ensure health information is being protected. These recommendations range from complex, structural adaptations in accordance with information technology departments, simulations of and education on cyberattacks and cybersecurity, to less complex actions, like making sure policies are available in non-digital copies, passwords are regularly renewed, and requiring multifactor authentication access to certain devices whenever possible, to more simple measures like not leaving workstations unattended when open to patient data, not sharing your passwords and treating patient data as it was your own. Even recommendations to incorporate cybersecurity in the AmSECT standard or guideline is mentioned in the manuscript [7]. Congratulations to Kara Lung (who also just happened to have been appointed to our journal’s Editorial Board this year!), for her fine work.

I hope you will enjoy the March 2026 edition of the Journal of ExtraCorporeal Technology, and may your brain continue to witness neuroplasticity, by browsing and reading the many contributions featured within.

## References

[1] Wu H, Yan H, Yang Y, et al. Occupational neuroplasticity in the human brain: A critical review and meta-analysis of neuroimaging studies, Front Hum Neurosci. 2020;14:215. 32760257 10.3389/fnhum.2020.00215 PMC7373999

[2] Gazerani P. The neuroplastic brain: current breakthroughs and emerging frontiers. Brain Res., 2025;1858:149643. 40280532 10.1016/j.brainres.2025.149643

[3] May A. Experience-dependent structural plasticity in the adult human brain. Trends Cogn Sci. 2011;15(10):475–482. 21906988 10.1016/j.tics.2011.08.002

[4] Taubert M, Draganski B, Anwander A, et al. Dynamic properties of human brain structure: learning-related changes in cortical areas and associated fiber connections. J Neurosci. 2010;30(35):11670–11677. 20810887 10.1523/JNEUROSCI.2567-10.2010 PMC6633410

[5] Leigh P, Harman E, Husain SA. Perfusion strategy and surgical repair of Type A interrupted aortic arch and Type 1 aortopulmonary window: a case report, J Extra Corpor Technol. 2025;58(1):85–89. 10.1051/ject/2025052. PMC12984032 41823494

[6] Che Morales JL, Vargas Mendoza GK. Tracheoesophageal fistula repair with veno-venous extracorporeal support and Montgomery T-tube: a case report. J Extra Corpor Technol. 2025;58(1):90–94. 10.1051/ject/2025055. PMC12984033 41823495

[7] Lung K. Cybersecurity as it relates to perfusion. J Extra Corpor Technol. 2025;58(1):3–18. 10.1051/ject/2025064. PMC12984037 41823484

